# Management of Patients With Legg-Calvé-Perthes Disease at a Single Center in Jeddah, Saudi Arabia

**DOI:** 10.7759/cureus.26262

**Published:** 2022-06-23

**Authors:** Majed N Al-Osaimi, Amr A Alsubaihi, Abdul-Aziz A Basaqr

**Affiliations:** 1 Surgery Department, King Abdullah International Medical Research Centre, King Saud bin Abdul-Aziz University for Health Sciences, Jeddah, SAU; 2 Associate Consultant Orthopedic Surgeon, Surgery Department, King Abdullah Medical Complex, Jeddah, SAU; 3 Orthopedics, Security Forces Hospital-Makkah, Makkah, SAU

**Keywords:** saudi arabia, physical therapy, orthotics, legg-calvé-perthes disease, children

## Abstract

Background: Legg-Calvé-Perthes disease (LCPD) is an idiopathic pediatric hip disorder associated with avascular necrosis of the femoral head. Although there is no standardized and optimal treatment protocol for patients with LCPD, there are three primary treatment strategies: symptomatic treatment, nonsurgical containment using orthotic devices, and surgical containment.

Objective: This study aimed to describe the demographic characteristics, management and outcome of pediatric patients with LCPD at our center between 2005 and 2015.

Material and Methods: In this retrospective study, 23 patients with LCPD who represented all patients with LCPD treated at King Abdul-Aziz Medical City, Jeddah, Saudi Arabia between 2005 and 2015 were enrolled. Their demographic and clinical characteristics, treatment methods, and outcomes were evaluated.

Results: Descriptive statistics showed that most of the patients were males (87.0%), with a mean age of 7.1±2.4 years. None of the patients had a family history of LCPD. Approximately two-thirds of the patients reported hip pain as the chief concern. LCPD was observed in the left hip in 60.9% of patients, the right hip in 21.7% of patients, and both hips in 17.4% of patients. Approximately half of the patients (55.3%) were treated with physiotherapy, and 10.6% were treated with orthotics. Despite that, 10 patients (43.4%) required surgical management after the conservative approach, and six of them underwent pelvic Salter innominate osteotomy.

Conclusion: These results highlight the experience of a single center in managing patients with LCPD. Treatment was different based on patient age; non-surgical treatment, mainly physiotherapy, was predominant in younger children. However, regardless of the type of treatment, the earlier interventions have proven to provide better outcomes in patients with this health condition.

## Introduction

Legg-Calvé-Perthes disease (LCPD) is a pediatric hip disease of unknown cause that may lead to avascular necrosis (AVN) of the femoral head [1,2] and was initially described in 1910 [3]. It is the main form of hip disease in children, with an incidence rate of 2-294 per 1,000,000 children [2]. Despite the many studies that have been conducted, the etiology and optimal treatment remain unclear [4,5].

Researchers have indicated that the classification of LCPD corresponds to the disease stage, outcome, and predictors of outcome [3]. Understanding the disease’s pathophysiology helps identify four progression stages through radiography, including initiation, fragmentation, re-ossification, and healing [4]. Outcomes have also been classified radiographically based on the femoral head shape and joint congruency [3]. Traditional radiography is simple but has limited ability to display the cartilaginous mold during growth. However, it has been replaced by arthrography, which is better at detecting alterations in the shape of the femoral head and extrusion of the femoral head [5]. Moreover, the site of involvement of the femoral head as seen on anteroposterior and lateral radiographs is considered one of the patient outcomes [3]. In addition, the size of the subchondral fracture on lateral radiographs may be a good indicator of the degree of involvement of the femoral head [3,5]. Subsequently, outcomes have been linked to the height of the lateral column of the femoral epiphysis [3,5].

In the context of treatment, there is strong evidence that early intervention has the most favorable outcomes [4]. Overall, the treatment aims to reduce hip pain and hip stiffness and maintain hip mobility. This helps the femoral head to recover, grow, and attain good shape [4]. Although there is no standardized and optimal treatment protocol for patients with LCPD, there are three primary treatment strategies: symptomatic treatment, nonsurgical containment using orthotic devices, and surgical containment [4]. Surgical intervention is indicated for patients with severe disease, including deformation of the femoral head and subluxation of the epiphysis [6]. However, despite the frequent use of such interventions for LCPD, there is limited literature from Saudi Arabia. The present study aimed to describe the demographic characteristics, management and outcome of LCPD among pediatric patients who were treated at our center between 2005 and 2015.

## Materials and methods

Study design, population and study setting

This study was a retrospective cross-sectional study. All patients included in this study were aged below 15 years, had LCPD, and visited the pediatric orthopedic department at King Abdul-Aziz Medical City, Jeddah, Saudi Arabia, between 2005 and 2015. Patients with other causes of AVN were excluded.

Data collection methods

The data collection sheet for this study was filled by the authors of the present study using information from the medical records database, which comprised two sections. The first section included the following information: age at diagnosis, date of diagnosis, and gender. The second section included the following clinical data: medical history which included history of breech presentation, oligohydramnios, cesarean section, neonatal intensive care unit (NICU) admission, family history of the same disease, consanguinity, chief complaint and associated symptoms, trauma, hip fracture, past medical and surgical history, and treatment modalities whether conservative, surgical, or both and their outcomes.

Study plan

This study planned to recruit pediatric patients who fulfilled the inclusion criteria between 2005 and 2015. Data collection began after obtaining permission from the ethical and scientific committee of King Abdul-Aziz Medical City with approval reference number RJ16/068/J and date 20-02-2021. We scrupulously guarded the confidentiality of the participants' data throughout the study. Prior to data collection, the participants provided informed consent for study participation and publication of data.

Data management and statistical analysis

The SPSS statistical software package for Windows (version 20.0; IBM Corp., Armonk, NY, USA) was used for data entry and statistical analysis. Quality control was performed at the coding and data entry stages. Data are presented using descriptive statistics in the form of frequencies and percentages for qualitative variables and means and standard deviations for quantitative variables.

## Results

General characteristics of patients with Legg-Calvé-Perthes disease

During the study period, 23 patients (27 hips) had LCPD. These 23 patients comprised 20 (87.0%) males and three (13.0%) females. The age range was four to 14 years, and the mean age was 7.1±2.4 years. Of these patients, 16 (69.6%) were younger than eight years, and seven (30.4%) were older than eight years. Eight (30.8%) patients had a history of trauma, and nine (39.1%) had a previous medical illness; however, none had a previous history of surgery. Only one patient (14.3%) out of seven was born by cesarean section; however, none were born by breech delivery. None of these children had oligohydramnios exposure, required NICU admission, or had a family history of LCPD; however, two (8.7%) reported consanguinity between first cousins (Table 1).

**Table 1:** Characteristics of patients with Legg-Calvé-Perthes disease (n=23) SD, standard deviation; NICU, neonatal intensive care unit.

Characteristics	Frequency	Percent (%)
Age		
Range	4-14 years	
Mean ± SD	7.1±2.4 years	
Gender		
Male	20	87
Female	3	13
History of trauma		
Yes	8	30.8
No	11	47.8
Unknown	4	17.4
History of hip fracture		
No	18	78.3
Unknown	5	21.7
Previous medical history		
Yes	9	39.1
No	14	60.9
Previous surgical history		
No	16	69.6
Unknown	7	30.4
Breech birth		
No	5	21.7
Unknown	18	78.3
Oligohydramnios		
No	5	21.7
Unknown	18	78.3
Cesarean section		
Yes	1	4.3
No	6	26.1
Unknown	16	69.6
NICU admission		
No	5	21.7
Unknown	18	78.3
Family history		
No	7	30.4
Unknown	16	69.6
Consanguinity		
Yes	2	8.7
No	2	8.7
Unknown	19	82.6

Clinical characteristics and outcome of the intervention 

**Figure 1:**
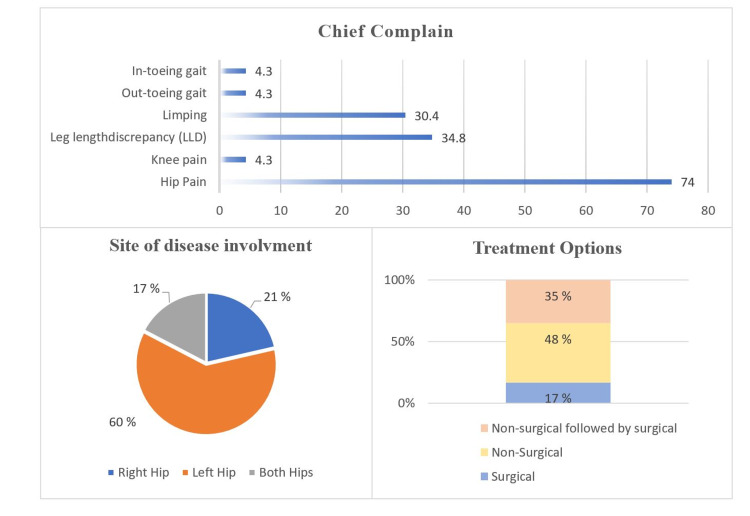
Clinical History of patients with Legg-Calvé-Perthes disease (n=23)

**Figure 2:**
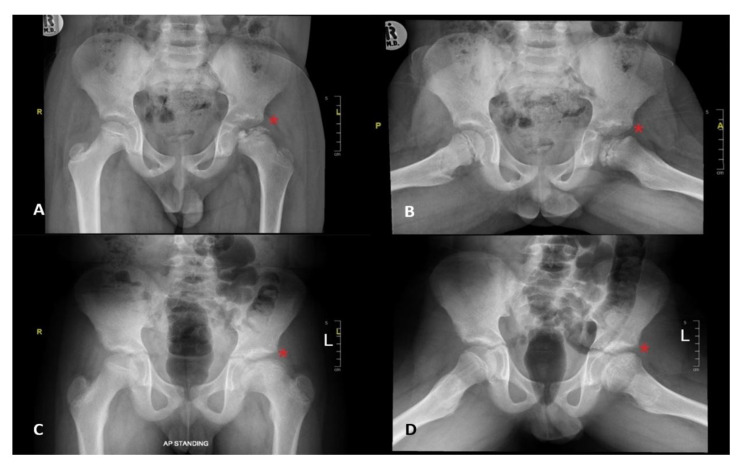
Example of pelvic x-rays of a patient with Legg-Calvé-Perthes disease aged younger than eight years before (A,B) and after (C,D) nonsurgical treatment.

**Figure 3:**
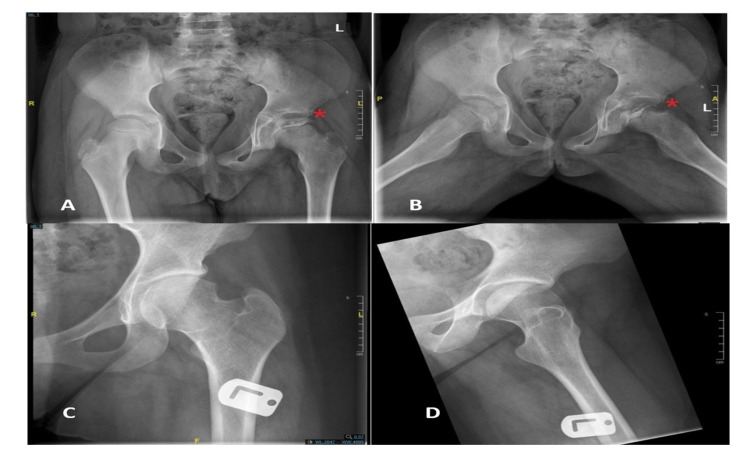
Example of pelvic x-rays before (A, B) and after (C, D) surgical treatment of patient with Legg-Calvé-Perthes disease aged younger than eight years.

**Figure 4:**
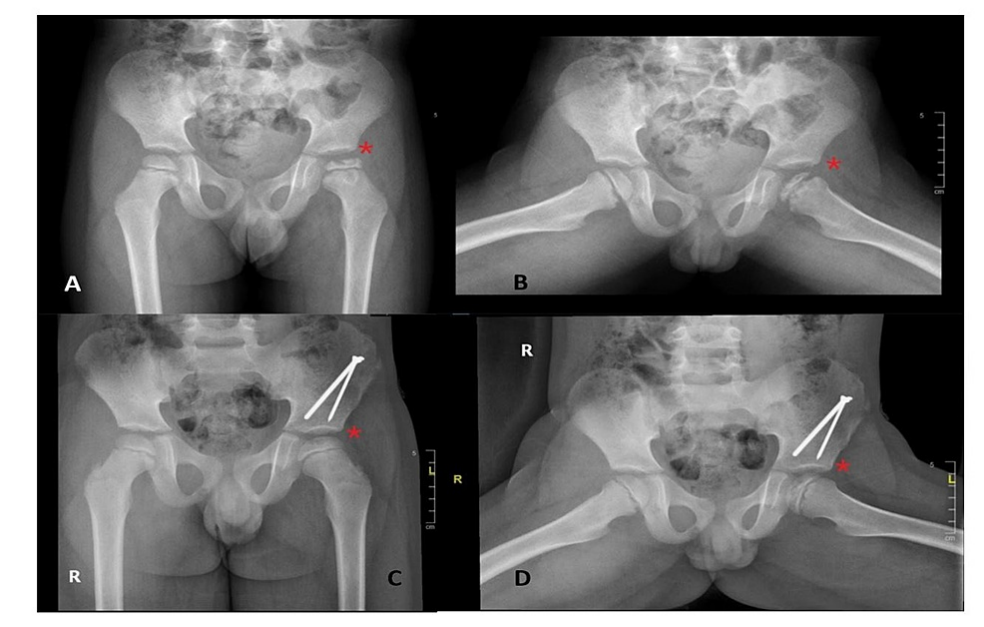
Example of pelvic x-rays of one patient with Legg-Calvé-Perthes disease older than eight years before (A,B) and after (C,D) Non-surgical treatment

**Figure 5:**
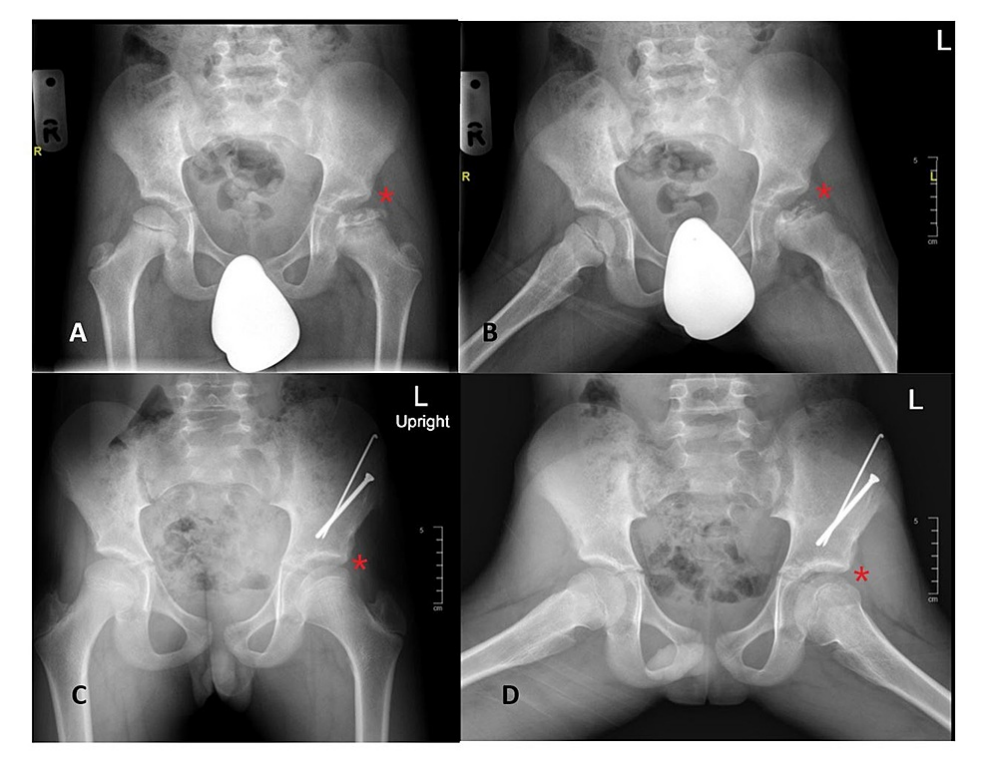
Example pf pelvic x-rays of one patient with Legg-Calvé-Perthes disease older than eight years before (A,B) and after (C,D) surgical treatment

Clinically, Figure 1 shows that 74% of the patients reported hip pain as the chief concern, followed by eight (34.8%) patients reporting leg length discrepancy and seven (30.4%) patients reporting limping as the chief concern. LCPD involved the left hip in 14 (60.9%) patients, right hip in five (21.7%) patients, and both hips in four (17.4%) patients. Twelve patients (44.4%) were categorized as having group C lateral pillar classification. Eleven (47.8%) patients underwent nonsurgical treatment (Figures 2, 3); eight (34.8%) initially received nonsurgical treatment, followed by surgery, and four (17.4%) underwent surgical treatment directly (Table 2, Figures 4, 5). Twelve patients (52.2%) were managed surgically, and half of them underwent pelvic Salter innominate osteotomy (Table 2). 

**Table 2:** Surgical treatment characteristics of patients with Legg-Calvé-Perthes disease

Characteristic	Frequency(n)	Percent (%)
Surgical management		
Surgery after nonsurgical management	8	34.8
Direct surgical management	4	17.4
Type of surgery		
Pelvic Salter innominate osteotomy	7	58
Femoral varus derotation osteotomy	1	8.3
Surgical dislocation hip reduction osteotomy, osteochondroplasty	1	8.3
Open reduction cheilectomy of femoral head and capsulotomy and Petri cast	1	8.3
Manipulation under anesthesia and casting and tenotomy later for total hip replacement	1	8.3
Intertrochanteric femoral valgus osteotomy and adductor tenotomy	1	8.3
Lateral pillar classification before surgery		
Group A	4	30.3
Group B	4	30.3
Group B/C	0	0
Group C	4	30.3
Involvement of the contralateral site		
Yes	3	13.0
No	18	74.0
Unknown	2	13.0

**Figure 6:**
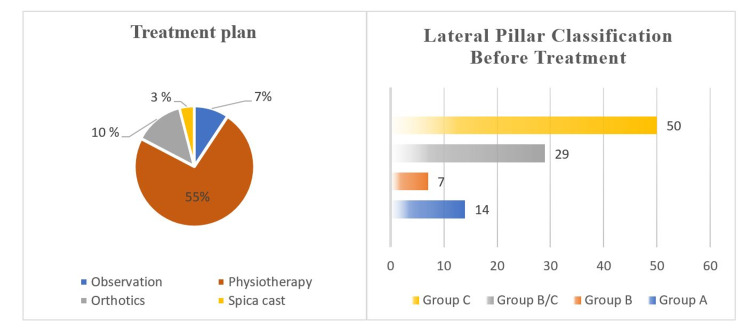
Treatment plan for the patients (n=23)

On the other hand, 19 patients were initially treated non-surgically, and among them, 16 (84.2%) were treated with physiotherapy, including range of motion exercises and non-weight-bearing activities (Figure 6). Three patients (15.7%) were treated with orthotics (abductor brace), and two (10.5%) patients were only under observation.

Most patients showed radiologic improvements, as well as improvements in the range of movement, with patients who underwent surgical treatment showing greater improvement than those treated without surgery (Table 2). Moreover, all patients reported significant reductions in pain following both surgical and non-surgical treatments.

## Discussion

Children diagnosed with LCPD have a high risk of early osteoarthritis as adults. Methods of preventing this risk include surgical and non-surgical modalities that mainly aim to restore a contained spherical femoral head [2]. Therefore, the present study described the experience of pediatric patients with LCPD who were treated at a single center. However, most patients (87%) in the present study were males, which is consistent with previous studies showing a male predominance in patients diagnosed with LCPD in the USA (90%) [7] and Italy (68.6%) [8].

Clinically, patients with LCPD usually present with pain, limited hip movement, and gait disturbance [2]. More than half of the patients reported hip pain as their chief concern in the present study, followed by leg length discrepancy and limping.

For prediction purposes, orthopedic surgeons use the recommended classification system to indicate functional outcomes and recommend treatment. However, an effective classification system should be reproducible, depend on indicators related to the disease process, and allow the grouping of similar hips, thereby enabling comparisons of treatment outcomes [3]. Although there are several proven advantages of using a classification system, some patients experience good outcomes in the absence of an intervention, whereas others require surgical or non-surgical treatment [9,10]. The lateral pillar classification system, which depends on the height of the lateral part of the capital femoral epiphysis during the fragmentation stage of the disease, correlated with the outcome [11,12]. It divides the femoral head into three pillars: the central, lateral, and medial pillars, and classifies LCPD into four groups, with group A defined as those with no loss of height or density in the lateral pillar; groups B and C, <50% and >50% loss of the original height in the lateral pillar, respectively; and group B/C, the border at which there are three distinct types [13,14]. Of the 23 patients in the present study, six (22.2%) were classified as having group A, five (18.5%) as having group B, four (14.8%) as having group B/C, and 12 (44.4%) as having group C LCPD. LCPD involved the left hip in 14 (60.9%) patients, the right hip in five (21.7%) patients, and both hips in four (17.4%) patients. This is consistent with the results of a previous study that reported bilateral LCPD in only 12% of the cases [15].

The type of treatment depends on two main factors: patient age and the extent of the femoral head involvement [2]. Children aged eight or under showed better responses to treatment and better outcomes than older children [6,16]. This is likely due to the time allowed for the femoral head to remodel before reaching skeletal maturity, which is longer in younger children than in older children [17,18]. Moreover, older children weigh more, thereby increasing mechanical stress on the hips [17,18]. Similar findings were observed in the present study, as more than half of the patients aged eight or under were treated non-surgically, whereas more than half of the patients aged more than eight years required both non-surgical and surgical management.

Symptomatic therapy has been recommended for patients of all ages with group A LCPD and patients aged under seven years with group B LCPD [14]. However, surgical treatment has been recommended for patients of all ages with group C LCPD and patients aged over six years with group B LCPD.

The results of the current study showed that physiotherapy was the most common non-surgical treatment. Physiotherapy included range of motion and non-weight-bearing exercises followed by orthotics. A study reported that treatment with Scottish Rite orthosis for 12 months did not reduce the power applied to the hip joint but reduced the angle of hip joint abduction while walking [19]. Abduction plaster casts were found to be successful in 60% of patients with a mean age of seven years [20], whereas abductor brace treatment showed good results in 63% of patients with a mean age of 8.6 years [21]. 

The primary surgical method was pelvic Salter innominate osteotomy in the current study, which showed radiological and range of movement improvements in 50% of patients. In agreement with this, previous studies showed that femoral osteotomy surgery improved outcomes in 58% of patients [22], and innominate osteotomy improved outcomes in 15 (56%) of 27 patients [23]. While Lloyd-Roberts et al. reported good results in 44.4%, fair in 22.2%, and poor in 33.4% of patients aged over six years, femoral osteotomy yielded good, fair, and poor results in 44.4%, 22.2%, and 33.4% of patients aged over six years, respectively [6,23]. A meta-analysis showed that outcomes were better after pelvic procedures compared with those after femoral procedures [2].

Lastly, the present study has several limitations, including its retrospective design, which may have resulted in a loss of information. Moreover, the small number of patients, combined with various pathologies and treatments, limited the ability to generalize the results of this study among this population. Additionally, many patients were lost to follow-up, thus limiting our ability to determine the long-term effects of treatment. Finally, the range of motion and patient scoring systems have not been adequately documented. 

## Conclusions

The present study shows the experience of a single center in managing patients with LCPD. A detailed clinical evaluation is essential and should always be correlated with radiological assessment. The prognosis of LCPD regressed to different prognostic variables, included age at presentation and the femoral head sphericity, and congruence. Designing a public health program may help to increase the awareness and will encourage parents to visit the clinic in the early stages of the disease.
